# dsRNA-induced condensation of antiviral proteins modulates PKR activity

**DOI:** 10.1073/pnas.2204235119

**Published:** 2022-08-08

**Authors:** Giulia A. Corbet, James M. Burke, Gaia R. Bublitz, Jian Wei Tay, Roy Parker

**Affiliations:** ^a^Department of Biochemistry, University of Colorado, Boulder, CO 80309;; ^b^BioFrontiers Institute, Boulder, CO 80309;; ^c^Howard Hughes Medical Institute, Chevy Chase, MD 20815-6789

**Keywords:** dsRNA, PKR, condensate

## Abstract

Mammalian cells respond to dsRNA in multiple manners. One key response to dsRNA is the activation of PKR, an eIF2α kinase, which triggers translational arrest and the formation of stress granules. However, the process of PKR activation in cells is not fully understood. In response to increased endogenous or exogenous dsRNA, we observed that PKR forms novel cytosolic condensates, referred to as dsRNA-induced foci (dRIFs). dRIFs contain dsRNA, form in proportion to dsRNA, and are enhanced by longer dsRNAs. dRIFs enrich several other dsRNA-binding proteins, including ADAR1, Stau1, NLRP1, and PACT. Strikingly, dRIFs correlate with and form before translation repression by PKR and localize to regions of cells where PKR activation is initiated. We hypothesize that dRIF formation is a mechanism that cells use to enhance the sensitivity of PKR activation in response to low levels of dsRNA or to overcome viral inhibitors of PKR activation.

## Significance

The presence of dsRNA in the cytosol is a marker of infection and elicits an immune response. One aspect of this immune response is the activation of the eIF2α kinase PKR, which results in translational reprogramming and stress granule formation. Here, we show that dsRNA induces the formation of a novel condensate by PKR that is distinct from other known ribonucleoprotein assemblies. These results challenge prior observations that PKR is recruited to stress granules and suggest that the condensation of PKR may be a mechanism that cells use to modulate PKR activation.

Mammalian cells initiate cell-autonomous innate immune responses upon the detection of double-stranded RNA (dsRNA), triggering a signaling cascade (reviewed by Jensen and Thomsen, 2012 [1]). This signaling cascade is initiated by numerous dsRNA sensors in the cell, also known as pattern recognition receptors (PRRs) (reviewed by Takeuchi and Akira, 2010 [2]). One of these PRRs is protein kinase R (PKR), which binds to dsRNA via its N-terminal RNA-binding domains and forms homodimers (3, 4). PKR dimerization on dsRNA results in autophosphorylation, leading to the full activation of PKR catalytic activity (4–6). Activated phospho-PKR (p-PKR) phosphorylates the eukaryotic translation initiation factor eIF2α on serine 51 (p-eIF2α), which inhibits canonical AUG-dependent translation initiation (7, 8). This process shuts off bulk translation to reduce viral gene expression while promoting the expression of select host mRNA transcripts involved in the integrated stress response (9).

The inhibition of translation by PKR results in the disassociation of most cellular mRNAs from ribosomes. A fraction of these nontranslating mRNAs condenses into cytoplasmic ribonucleoprotein complexes (RNPs) called stress granules (SGs), which are enriched with large RNAs and several RNA-binding proteins, including G3BP1/2, TIA-1, UBAP2L, and the poly(A)-binding protein (PABP) (10, 11). Various studies have reported the recruitment of dsRNA sensors, including PKR, MDA-5, RIG-I, and OAS, to SGs assembled in response to dsRNA, viral infection, G3BP1 overexpression, or oxidative stress (12–20). These reports propose that interactions between SG proteins modulate the activation of dsRNA sensors to regulate the dsRNA response. For example, PKR localization to SGs was proposed to promote phosphorylation of eIF2α (16), and RIG-I localization to SGs was proposed to promote the RIG-I/MAVS/IRF3 signaling pathway (14, 15, 17).

While SGs are presumed to promote the antiviral response, we recently showed that activation of the 2′,5′-oligoadenylate synthetase (OAS)/RNase L antiviral pathway inhibits the assembly of canonical SGs by degrading cellular RNAs (21, 22). Moreover, RNase L (RL)–mediated RNA decay promotes the formation of SG-like RNP complexes called RL-dependent bodies (RLBs), which contain many SG-enriched RNA-binding proteins, including G3BP1/2, caprin-1, and PABP, but are distinct in their biogenesis, morphology, and composition in comparison to SGs.

Here, we sought to determine how the localization and function of PKR occurs relative to cytoplasmic RNP granules. Surprisingly, in contrast to previous studies, we did not observe that PKR localized to SGs. Instead, we observed that PKR forms novel foci in response to foreign or endogenous dsRNA that are distinct from both RLBs and SGs. These foci, which we have called dsRNA-induced foci (dRIFs), contain dsRNA and various dsRNA-binding proteins, including PKR, ADAR1, Stau1, DHX9, NLRP1, and PACT. dRIFs form before or concurrently with PKR-mediated translational repression and localize to the region of the cell where translation repression initiates. This demonstrates that dRIFs temporally and spatially correlate with PKR activation. Furthermore, we observed that dRIF assembly is independent of PKR, indicating that these are not PKR-driven condensates. These findings identify a dsRNA-protein condensate that forms during the innate antiviral response and may modulate the activity of PKR.

## Results

### PKR Forms Distinct Foci during dsRNA Stress.

**Fig. 1.**
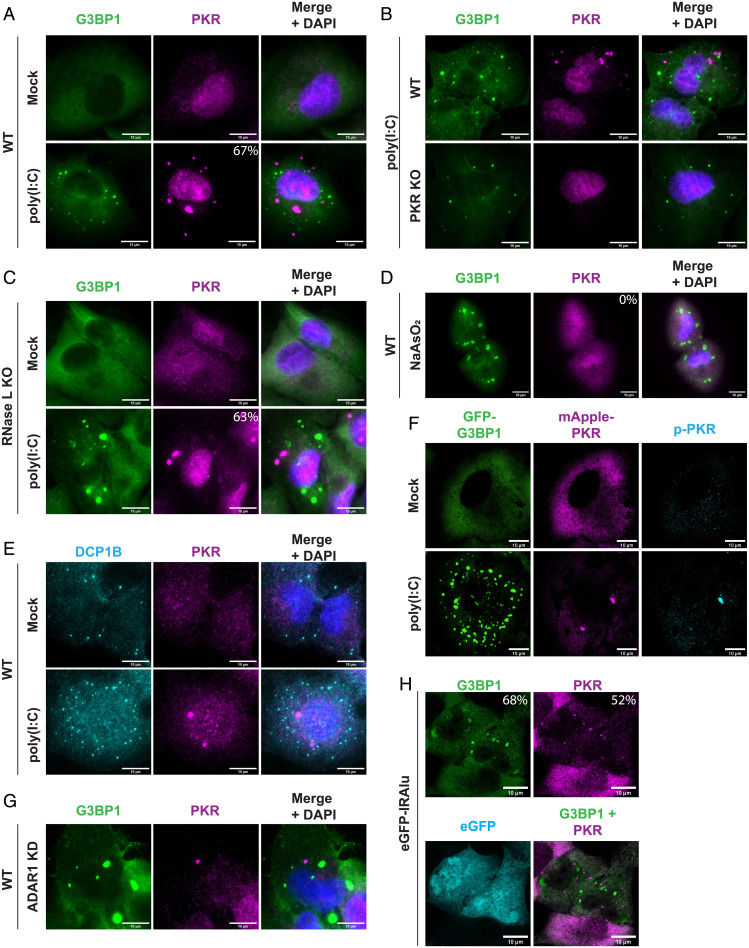
PKR forms distinct foci during dsRNA stress. (*A*) IF for PKR and G3BP1 in WT A549 cells transfected with 500 ng/mL poly(I:C) for 4 h. Nuclei stained with DAPI. Percentage of cells with PKR foci indicated. A total of 591 cells analyzed from 7 independent experiments. SE, 3.6%. (*B*) IF for PKR and G3BP1 in WT and PKR KO A549 cells transfected with poly(I:C). (*C*) IF for PKR and G3BP1 in RL KO A549 cells transfected with poly(I:C). Percentage of cells with PKR foci indicated. A total of 1,379 cells analyzed from 7 independent experiments. SE, 2.5%. (*D*) IF for PKR and G3BP1 in A549 cells treated with 500 μM NaAsO_2_ for 1 h. (*E*) IF for DCP1B and PKR in A549 cells transfected with poly(I:C). (*F*) IF for p-PKR in PKR/RL KO A549 cells expressing mApple-PKR and GFP-G3BP1 and transfected with 500 ng/mL poly(I:C) for 4 h. (*G*) IF for PKR and G3BP1 in ADAR1 small interfering RNA (siRNA)-treated U-2 OS cells. (*H*) IF for PKR and G3BP1 in RL KO A549 cells transfected with eGFP-IRAlu reporter plasmid. A total of 166 cells analyzed across 4 independent experiments. SE for percentage of cells with dRIFs, 5.1%. SE for percentage of cells with SGs, 4.1%. (All scale bars, 10 μm.)

To test whether we could reproduce the localization of PKR to SGs, we stained cells for PKR and the SG marker G3BP1 after transfection of the synthetic dsRNA poly(I:C) into wild-type (WT) A549 cells (Fig. 1*A*). Surprisingly, we did not observe colocalization between PKR and G3BP1; instead, PKR formed distinct foci in 67% of cells (Fig. 1*A*). In the remaining 33% of cells, PKR did not form visible foci and still did not colocalize with G3BP1 assemblies. Similar results were observed in WT U-2 OS cells (see *SI Appendix*, Fig. S1*A*). To validate that we were visualizing PKR, we performed the same poly(I:C) transfection and staining in PKR knockout (KO) A549 cells (23) (*SI Appendix*, Fig. S1*B*). While some nonspecific antibody staining can be observed in the nuclei of PKR KO cells, we did not observe any PKR assemblies in the PKR KO cells (Fig. 1*B*), validating that the foci we observed in the WT cells contained PKR.

We could not directly rule out PKR localization to SGs in the above experiment because WT A549 cells form small G3BP1 puncta known as RLBs instead of SGs after poly(I:C) transfection due to widespread RNA degradation by RL (Fig. 1*A*) (21, 22). To directly ask whether PKR would localize to SGs, we examined the subcellular localization of PKR and G3BP1 after poly(I:C) transfection into RL KO A549 cells, which form SGs instead of RLBs due to the absence of RL (22). We observed that PKR formed distinct foci in the cytoplasm in 63% of cells and did not localize to SGs (Fig. 1*C*). Similar results were observed in RL KO U-2 OS cells (*SI Appendix*, Fig. S1*C*). We also did not observe PKR enrichment in SGs under arsenite stress by immunofluorescence (IF) or using an mApple-tagged PKR construct (Fig. 1*D* and *SI Appendix*, Fig. S1*D*), suggesting that PKR does not localize to SGs in multiple different stresses. Supporting this observation, another recent study also found distinct PKR foci during dsRNA stress (24).

One possibility is that PKR foci form due to PKR localizing upon poly(I:C) transfection to P-bodies, which are cytoplasmic RNP granules containing nontranslating mRNAs and the RNA decay machinery (25). To test this possibility, we stained A549 cells for PKR and the P-body marker DCP1B. We did not observe colocalization between PKR foci and DCP1B-marked P-bodies (Fig. 1*E*). This demonstrates that PKR forms novel, distinct foci. However, in U-2 OS cells, we did observe that occasionally PKR assemblies are adjacent to P-bodies (*SI Appendix*, Fig. S1*E*), suggesting there may be some interaction between PKR assemblies and P-bodies. Interestingly, another recent report observed some association between PKR assemblies and P-bodies by live-cell imaging (24). They observed de-mixing of PKR and DCP1A assemblies shortly after coassembly, which may partially explain why our IF did not indicate colocalization between P-bodies and PKR foci several hours after poly(I:C) transfection.

A recent report described that cytosolic dsRNA induces condensation of the inflammasome protein NLRP6 (26). To test whether the PKR assemblies we observed are related to NLRP6 condensates, we transfected a GFP-NLRP6 fusion protein into a cell line expressing mApple-PKR. Upon poly(I:C) transfection, we observed that mApple-PKR forms foci that do not recruit GFP-NLRP6 (*SI Appendix*, Fig. S1*F*). Only in less than 15% of transfected cells did we observe the overexpressed GFP-NLRP6 transiently forming assemblies, and these also did not recruit mApple-PKR (*SI Appendix*, Fig. S1*F*). These results indicate that PKR and NLRP6 form distinct assemblies in poly(I:C) stress, with PKR foci being more prevalent in A549 cells.

Poly(I:C) is a potent activator of PKR and induces the formation of PKR foci. To test whether PKR is active in these foci, we stained RL KO A549 cells expressing mApple-PKR and GFP-G3BP1 for p-PKR, the active form of PKR. Upon poly(I:C) transfection, we observed the enrichment of p-PKR in mApple-PKR–marked foci (Fig. 1*F*), although the relative amount of p-PKR varied between dRIFs. This observation indicates that active PKR is enriched in PKR foci.

We next examined whether the formation of PKR foci was a common feature of dsRNA stress or whether it was restricted to exogenously transfected poly(I:C). We and others have previously observed that depletion of the dsRNA-modifying protein ADAR1 triggers PKR activation and SG assembly in 5 to 25% of cells (27, 28), presumably due to increased endogenous dsRNA from the lack of RNA editing. Thus, we tested whether ADAR1 knockdown (KD) cells induced the formation of PKR foci. We observed that in rare instances (<1% of cells) in WT U-2 OS cells, ADAR1 KD triggered the formation of PKR foci in the same cells that had SGs. This suggests that PKR foci formation is linked to PKR activation (Fig. 1*G* and *SI Appendix*, Fig. S1*G*). This observation demonstrates that endogenous dsRNAs can trigger PKR foci formation. However, since some ADAR1 KD cells form SGs without the formation of visible PKR foci, these foci, at least at the scale able to be visualized on a light microscope, are not a requirement for PKR activation.

In a second experiment to examine whether endogenous dsRNAs can induce PKR foci formation, we used a reporter plasmid expressing enhanced green fluorescent protein (EGFP) with an inverted *Alu* repeat (*IRAlu*) in the 3′ untranslated region (UTR) (29). When transcribed, the *IRAlu* repeat forms ∼300 bases of contiguous dsRNA structure, which should bind and activate PKR. Upon transient transfection of the reporter plasmid, we observed that 52% of EGFP^+^ cells formed PKR foci and 68% had SGs (Fig. 1*H*). This provides a second observation that endogenous dsRNAs can induce PKR foci formation and link the formation of these foci to PKR activation and translational repression.

Taken together, these results demonstrate that both endogenous and exogenous dsRNA induce the formation of PKR foci (dRIFs). Similar formation of PKR foci has also been observed in response to poly(I:C) transfection or measles infection (24).

### dRIFs Contain dsRNA.

**Fig. 2.**
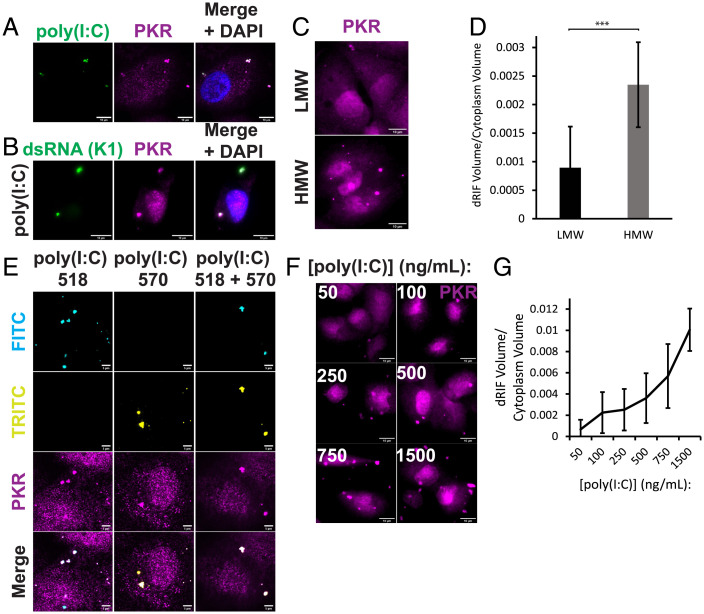
dRIFs contain dsRNA. (*A*) IF for PKR in poly(I:C)-518-transfected A549 cells. Nuclei stained with DAPI. (Scale bars, 10 μm.) (*B*) IF for PKR and dsRNA (K1 antibody) in A549 cells transfected with poly(I:C). (Scale bars, 10 μm.) (*C*) IF for PKR in A549 cells transfected with 500 ng/mL LMW or HMW poly(I:C). (Scale bars, 10 μm.) (*D*) Quantification of dRIF volume in (*C*). A total of 12 images from 2 independent experiments quantified. Error bars represent SDs. Unpaired 2-tailed *t* test, ***P < 0.001. (*E*) IF for PKR in poly(I:C)-518 and poly(I:C)-570-transfected A549 cells. White arrows indicate location of dRIFs. (Scale bars, 5 μm.) (*F*) IF for PKR in A549 cells transfected with 50 to 1,500 ng/mL poly(I:C) for 4 h. (Scale bars, 10 μm.) (*G*) Quantification of dRIF volume relative to cytoplasmic volume in (*F*). A total of 5 images quantified for each condition. The line represents the average and error bars represent the SDs of dRIF volume measured at indicated concentrations of poly(I:C).

Since either endogenous or exogenous dsRNA triggers the formation of dRIFs, we transfected cells with fluorescently labeled poly(I:C) and asked whether dsRNA colocalized with PKR (Fig. 2*A*). We observed that the poly(I:C) did colocalize with PKR. We further confirmed this colocalization of poly(I:C) with PKR by staining for dsRNA with the dsRNA-specific K1 antibody (Fig. 2*B*). In addition, live cell imaging of a cell line expressing mApple-PKR transfected with fluorescently labeled poly(I:C) demonstrated recruitment of PKR to dsRNA foci upon poly(I:C) entering the cell (*SI Appendix*, Fig. S1*H*). This suggests a model in which poly(I:C) functions as a scaffold for the formation of higher order PKR assemblies.

If poly(I:C) is the scaffold for dRIF formation, then the length of poly(I:C) would be expected to influence the size and/or number of dRIFs observed, with longer dsRNAs being more prone to forming dRIFs due to increased valency for interactions. To address this question, we transfected cells with equal nanograms of low-molecular-weight (LMW) and high-molecular-weight (HMW) poly(I:C). Because equal quantities of poly(I:C) were used, any differences in foci size and/or number can be explained by the difference in length of the poly(I:C) molecules.

We observed that HMW poly(I:C) produces a larger volume of PKR foci than LMW poly(I:C) for the same mass of transfected dsRNA (Fig. 2*C* and *D*). This result suggests that poly(I:C) serves as a scaffold for dRIF formation and that longer poly(I:C), which presumably contains more binding sites, can seed larger foci formation. Similar results have been seen with increased interaction sites enhancing protein-based condensate formation (30), and are consistent with the observations that longer RNAs are more effective at enhancing RNA condensation in vitro (31) and in cells (11). Interestingly, no difference was observed in the phosphorylation of eIF2α upon transfection with equal doses of HMW or LMW poly(I:C), indicating that larger foci formation does not necessarily result in differences in PKR activation (*SI Appendix*, Fig. S1*I*).

In principle, these assemblies could be composed of one or multiple dsRNA molecules bound to many PKR molecules. To test whether multiple dsRNA molecules are present in these foci, we transfected A549 cells with two different colors of fluorescently labeled poly(I:C). If these assemblies are composed of only one molecule of dsRNA, then we should only ever observe one color of poly(I:C) in a single assembly. However, we observed assemblies that contained both colors of poly(I:C) (Fig. 2*E*), demonstrating that multiple molecules of dsRNA are present within each dRIF.

We wanted to test whether altering amounts of poly(I:C) had any effect on the formation of dRIFs. We transfected A549 cells with 50 to 1,500 ng/mL poly(I:C) and stained for PKR. We observed a dose-dependent effect on dRIF volume with increasing amounts of poly(I:C) (Fig. 2*F* and *G*), demonstrating that dRIF formation was dependent on the dsRNA concentration.

Titration of increased concentrations of poly(I:C) also resulted in a dose-dependent increase in p-PKR and p-eIF2α on a population level (*SI Appendix*, Fig. S1*J*). We also observed increased enrichment of p-PKR in poly(I:C)-marked dRIFs with increased concentrations of poly(I:C) (*SI Appendix*, Fig. S1*K*).

To determine whether other RNAs could be recruited to dRIFs, we examined whether poly(A)^+^ RNA, which is enriched in SGs and RLBs, is also enriched in dRIFs. Upon staining for poly(A)^+^ RNA, the SG/RLB marker G3BP1, and PKR, we observed that poly(A)^+^ RNA is not enriched in dRIFs, but is enriched in RLBs, which stain positive for G3BP1 (*SI Appendix*, Fig. S2*A*) (22). Together, these results suggest that the RNA content of dRIFs is primarily dsRNA.

Cycloheximide (CHX) is a translation inhibitor that traps mRNAs on ribosomes (32). mRNAs must be released from ribosomes to be recruited to SGs; thus, SG formation is inhibited by CHX (33, 34). Given that dRIFs are not enriched in poly(A)^+^ RNA, we would not expect dRIF formation to require mRNA release from ribosomes. To test this, we added 10 μg/mL CHX to RL KO A549 cells and transfected with 500 ng/mL poly(I:C) at the same time. After 2 h, dRIF and SG formation was assessed by IF for PKR and G3BP1. While CHX prevents the formation of poly(I:C)-induced SGs, PKR recruitment to foci was not affected by CHX treatment (*SI Appendix*, Fig. S2*B*), further confirming that nontranslating mRNAs are not a major constituent of dRIFs.

Together, these observations demonstrate that dRIFs are assemblies of PKR and dsRNA. Both the average length and quantity of dsRNA added alter the volume of dRIFs formed, suggesting that dsRNA acts as a scaffold for dRIF assembly.

### dRIFs Contain PKR-Interacting Proteins and dsRNA-Binding Proteins.

Given that dRIFs appear to be composed of dsRNA and at least one dsRNA-binding protein, PKR, we hypothesized that other dsRNA-binding proteins and PKR-interacting proteins may also enrich in dRIFs. Thus, we performed IF for candidate proteins and assessed their localization upon poly(I:C) treatment.

**Fig. 3.**
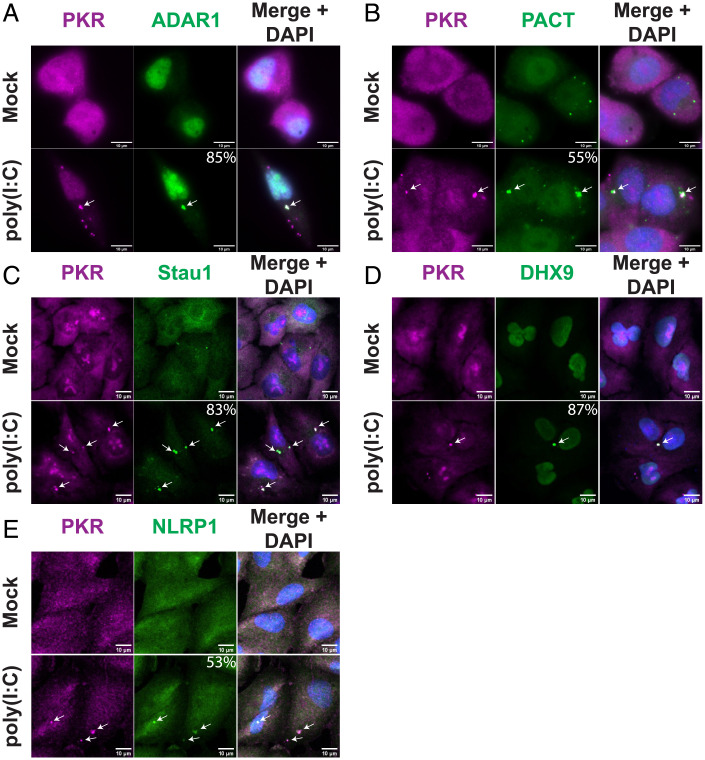
dRIFs contain dsRNA-binding proteins. (*A*) IF for PKR and ADAR1 in A549 cells transfected with poly(I:C). White arrows indicate colocalization. Percentage of PKR^+^ dRIFs enriched for ADAR1 indicated. A total of 119 foci analyzed from 5 independent experiments. SE, 5.1%. (*B*) IF for PKR and PACT in A549 cells transfected with poly(I:C). Percentage of PKR^+^ dRIFs enriched for PACT indicated. A total of 147 foci analyzed from 4 independent experiments. SE, 16.7%. (*C*) IF for PKR and Staufen (Stau1) in A549 cells transfected with poly(I:C). Percentage of PKR^+^ dRIFs enriched for Stau1 indicated. A total of 102 foci analyzed from 3 independent experiments. SE, 0.5%. (*D*) IF for PKR and DHX9 in A549 cells transfected with poly(I:C). Percentage of PKR^+^ dRIFs enriched for DHX9 indicated. A total of 69 foci analyzed from 4 independent experiments. SE, 3.3%. (*E*) IF for PKR and NLRP1 in A549 cells transfected with poly(I:C). Percentage of PKR^+^ dRIFs enriched for NLRP1 indicated. A total of 125 foci analyzed from 3 independent experiments. SE, 3.3%. (All scale bars, 10 μm.)

Upon staining for the dsRNA-binding and modifying enzyme ADAR1, we observed ∼85% of PKR^+^ foci were enriched for ADAR1 upon poly(I:C) treatment (Fig. 3*A*). Thus, ADAR1 is a component of dRIFs, although the ratios between ADAR1 and PKR varied between individual dRIFs.

Protein activator of PKR (PACT) is another dsRNA-binding protein that is activated upon stress and activates PKR by direct binding (35, 36). Staining for PACT revealed that it is punctate even in nonstressed conditions (Fig. 3*B*). Upon poly(I:C) treatment, the PACT foci persisted, and PACT was enriched in 55% of PKR^+^ foci (Fig. 3*B*). Live-cell imaging of a cell line expressing mApple-PACT and PKR-GFP validated the recruitment of both PKR and PACT to foci upon poly(I:C) transfection (*SI Appendix*, Fig. S2*C*). While mApple-PACT does not form foci in the absence of added dsRNA, unlike what was observed when staining for endogenous PACT (Fig. 3*B*), it is recruited to foci upon poly(I:C) transfection.

dsRNA-binding protein Staufen homolog 1 (Stau1) is a highly conserved dsRNA-binding protein with roles in RNA localization, stability, and translation (37–39). IF for Stau1 under mock conditions revealed that it is largely diffuse in normal conditions, with a few small puncta detected (Fig. 3*C*). Upon poly(I:C) transfection, Stau1 is enriched in 83% of PKR^+^ dRIFs (Fig. 3*C*).

DHX9, also known as RNA helicase A, is an essential dsRNA-binding protein with roles in RNA processing (40, 41). IF for DHX9 revealed that it is diffuse throughout the nucleus and cytoplasm during normal conditions, but upon poly(I:C) treatment, DHX9 is enriched in 87% of PKR^+^ dRIFs (Fig. 3*D*). The inflammasome protein NLRP1 was recently demonstrated to have dsRNA-binding activity (42) and appeared to be enriched in 53% of PKR^+^ foci (Fig. 3*E*).

Taken together, these results identify dRIFs as minimally containing dsRNA, PKR, PACT, ADAR1, Stau1, NLRP1, and DHX9. However, the enrichment of each of these dsRNA binding proteins varies, suggesting that there is variation in the composition of each dRIF. Moreover, there appears to be selectivity in the recruitment of dsRNA-binding proteins to dRIFs since we saw no enrichment of the dsRNA-binding proteins NLRP3, NLRP6, TLR3, RIG-I, and Tudor-SN in dRIFs (*SI Appendix*, Figs. S1*F* and S2*D*), although we cannot rule out that these proteins are present in dRIFs at levels similar to the bulk cytosol, or that the IF signal from these antibodies is insufficient to determine their localization.

### dsRNA Binding Enhances Protein Recruitment to dRIFs.

In principle, the recruitment of proteins to dRIFs could be dependent on dsRNA binding, as well as protein–protein interactions. To test the role of dsRNA binding for dRIF protein recruitment, we examined whether the dsRNA-binding ability of ADAR1 is necessary and/or sufficient for recruitment to dRIFs using previously generated truncation variants of the cytoplasmic isoform of ADAR1 (ADAR1 p150) (28). ADAR1 p150 is known to localize to SGs induced by arsenite by its N-terminal Z domains (28, 43). Thus, we asked whether various forms of ADAR1 p150 localize to dRIFs or SGs after poly(I:C) transfection.

**Fig. 4.**
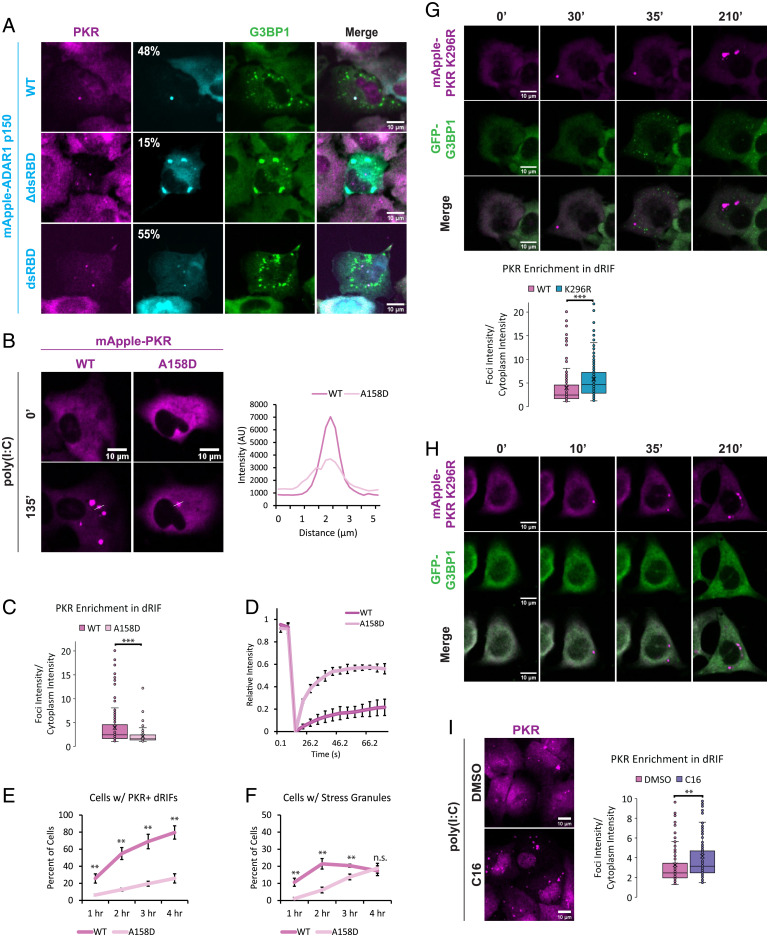
dsRNA-binding enhances protein recruitment to dRIFs. (*A*) IF for PKR and G3BP1 in RL KO A549 cells transiently transfected with mApple-ADAR1 p150 WT, ΔdsRBD, or dsRBD and transfected with poly(I:C). Percentage of PKR^+^ dRIFs enriched for ADAR1 p150 indicated. (*B*) A549 cells expressing mApple-PKR WT or A158D at 0 and 135 min after poly(I:C) transfection and a line scan showing the intensity profiles of foci. (*C*) Quantification of average enrichment of mApple-PKR WT and A158D in dRIFs over cytoplasm. At least 83 foci quantified per condition from 3 independent experiments. Unpaired 2-tailed *t* test, ***P < 0.001. (*D*) FRAP analysis of mApple-PKR WT and A158D dRIFs. Error bars represent SDs. Data represent average from 3 independent experiments. (*E*) Percentage of PKR/RL dKO A549 cells expressing GFP-G3BP1 and mApple-PKR WT or A158D with PKR^+^ dRIFs over time after poly(I:C) transfection. Data represent average from 3 independent experiments; error bars represent SDs. Unpaired 2-tailed *t* test, **P < 0.01. (*F*) Percentage of PKR/RL dKO A549 cells expressing GFP-G3BP1 and mApple-PKR WT or A158D with SGs over time after poly(I:C) transfection. Data represent average from 3 independent experiments; error bars represent SDs. Unpaired 2-tailed *t* test, **P < 0.01, n.s. = not significant. (*G*) PKR KO A549 cells expressing GFP-G3BP1 and mApple-PKR K296R transfected with 500 ng/mL poly(I:C) and imaged at 0, 30, 35, and 210 min after transfection. Quantification of mApple-PKR WT and K296R enrichment in dRIFs shown. At least 146 foci quantified from 2 or 3 independent experiments per condition. Unpaired 2-tailed *t* test, ***P < 0.001. (*H*) PKR/RL dKO A549 cells expressing GFP-G3BP1 and mApple-PKR K296R transfected with 500 ng/mL poly(I:C) and imaged at 0, 10, 35, and 210 min after transfection. (*I*) IF for PKR in RL KO A549 cells treated with C16 PKR inhibitor or DMSO for 24 h and transfected with 500 ng/mL poly(I:C) for 4 h. Quantification of PKR enrichment in dRIFs in DMSO and C16-treated A549 cells. At least 169 foci quantified per condition from 4 independent experiments. Unpaired 2-tailed *t* test, **P < 0.01. (All scale bars, 10 μm.)

We observed that in cells that formed both SGs and dRIFs in response to poly(I:C), ADAR1 p150 was recruited to dRIFs (Fig. 4*A*), which also validates the IF showing ADAR1 enrichment in dRIFs. Strikingly, we observed that the localization of ADAR1 p150 lacking its three dsRNA-binding domains (ΔdsRBDs) to dRIFs is greatly reduced, and it is enriched in SGs in the majority of instances upon poly(I:C) transfection (Fig. 4*A*). This suggests that recruitment of ADAR1 p150 to dRIFs is largely dependent on its dsRNA-binding activity. Consistent with this interpretation, a construct expressing only the three ΔdsRBDs of ADAR1 p150 localizes to dRIFs upon poly(I:C) transfection (Fig. 4*A*). These results demonstrate that ADAR1 ΔdsRBDs are both necessary and sufficient for efficient ADAR1 p150 recruitment to dRIFs, and in the absence of dsRNA binding, ADAR1 p150 can be recruited to SGs through its Z domains (28, 43).

To test the role of dsRNA binding in the recruitment of PKR to dRIFs, we used the A158D mutation, which abolishes the ability of PKR to bind to dsRNA (44). The overexpression of mApple-PKR WT or A158D triggered SG formation in WT A549 cells, likely due to aberrant PKR activation and inhibition of translation initiation (*SI Appendix*, Fig. S2*E*). This indicates that PKR is capable of autophosphorylation in the absence of dsRNA binding at high enough concentrations, which is consistent with previous results (45). Given this, we stably introduced mApple-PKR WT and A158D into PKR/RL double KO (DKO) A549 cells using lentiviral transduction (*SI Appendix*, Fig. S2*F*).

We observed that mApple-PKR A158D is recruited to dRIFs upon poly(I:C) transfection; however, its recruitment is diminished compared to WT PKR (Fig. 4*B*, *C*, and *E*). This observation suggests that PKR recruitment to dRIFs is a combination of binding dsRNA and protein–protein interactions between PKR and other dRIF proteins. Consistent with that model, fluorescence recovery after photobleaching (FRAP) analysis revealed that mApple-PKR A158D is much more mobile within dRIFs than WT, demonstrating that dsRNA binding contributes to the lack of mobility of PKR within dRIFs (Fig. 4*D*). Live-cell imaging revealed that mApple-PKR WT is recruited to dRIFs more quickly and in a higher proportion of cells than mApple-PKR A158D (Fig. 4*E*). This correlates to a higher proportion of mApple-PKR WT expressing cells having SGs, a readout of PKR-mediated translational shutoff, at earlier time points postpoly(I:C) transfection (Fig. 4*F*). By 4 h posttransfection, no difference in the proportion of cells with SGs is observed between WT- and A158D-expressing cells (Fig. 4*F*). Since in vitro studies demonstrated that PKR A158D is unable to be activated by dsRNA (46), these results show that in cells, PKR A158D may still be activated by dsRNA, possibly in part due to its recruitment to and concentration in dRIFs via protein–protein interactions that facilitate PKR molecules being in close enough proximity to autophosphorylate (*SI Appendix*, Fig. S2*F*). We do see some poly(I:C)-independent p-PKR signal in A158D cells (*SI Appendix*, Fig. S2*F*), potentially because this mutant is more highly expressed relative to endogenous PKR or due to nonspecific antibody binding to this mutant.

One possible role of dRIFs is to concentrate PKR into a condensate with dsRNA and thereby increase the rate of PKR autophosphorylation in *trans*. Once phosphorylated, the affinity of PKR for dsRNA decreases, making PKR more likely to dissociate from dRIFs (47). A prediction of this model is that the inhibition of PKR catalysis by mutation or with chemical inhibitors should lead to the prolonged persistence of PKR in dRIFs. To assess how PKR catalytic activity affects its recruitment to dRIFs, we generated a catalytically dead (K296R) mutant PKR and introduced it into PKR KO and PKR/RL DKO cells expressing GFP-G3BP1 (*SI Appendix*, Fig. S2*F*). Consistent with previous results (22), PKR KO cells expressing mApple-PKR K296R still form RLBs upon poly(I:C), because RL activation is not dependent on PKR activity (Fig. 4*G*). However, PKR/RL DKO cells expressing mApple-PKR K296R do not form SGs, because PKR-mediated phosphorylation of eIF2α is required for SG formation in response to poly(I:C) (Fig. 4*H*) (22). mApple-PKR K296R is more enriched in dRIFs than WT mApple-PKR, which is consistent with the nonphosphorylated form of PKR having a higher affinity for dsRNA (Fig. 4*G*). mApple-PKR K296R is still recognized by the p-PKR antibody, which we suggest is due to nonspecific antibody binding since this p-PKR signal is not dependent on poly(I:C) (*SI Appendix*, Fig. S2*F*), and SG induction is not observed in K296R-expressing cells (Fig. 4*H*).

C16 is a PKR inhibitor that binds to the adenosine triphosphate (ATP)-binding pocket of PKR, preventing autophosphorylation, but does not prevent dsRNA binding (48, 49). We tested whether the inhibition of PKR with C16 would alter PKR recruitment to foci upon poly(I:C) treatment. After 24 h of treatment with C16, A549 cells were transfected with poly(I:C) and stained for PKR. We observed an increase in the enrichment of PKR in dRIFs upon C16 treatment (Fig. 4*I*), which is, again, consistent with the unphosphorylated form of PKR having a higher affinity for dsRNA.

### dRIFs Predominantly Form before Translational Repression.

**Fig. 5.**
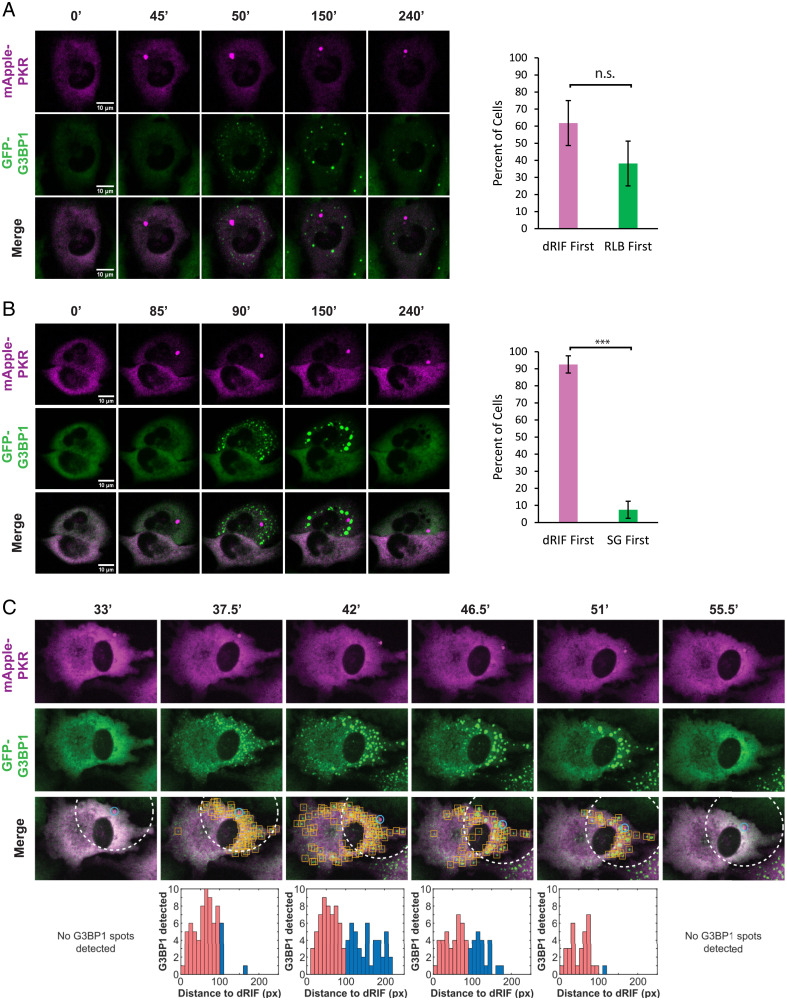
dRIFs predominantly form before translational repression. (*A*) PKR KO A549 cells expressing GFP-G3BP1 and mApple-PKR transfected 500 ng/mL poly(I:C) and imaged at 0, 45, 50, 150, and 240 min after transfection. Percentage of cells that form dRIFs first or RLBs first shown at right. Error bars represent SDs from 3 independent experiments. A total of 131 cells quantified from 3 independent experiments. n.s. = not significant. (Scale bars, 10 μm.) (*B*) PKR/RL DKO A549 cells expressing GFP-G3BP1 and mApple-PKR transfected with 500 ng/mL poly(I:C) and imaged at 0, 85, 90, 150, and 240 min after transfection. Percentage of cells that form dRIFs first or SGs first shown at right. Error bars represent SDs from 3 independent experiments. A total of 125 cells quantified from 3 independent experiments. ***P < 0.001. (Scale bars, 10 μm.) (*C*) Images showing asymmetric SG formation proximal to dRIF in PKR/RL DKO A549 cells expressing GFP-G3BP1 and mApple-PKR transfected with 500 ng/mL poly(I:C) and imaged at 33, 37.5, 42, 46.5, 51, and 55.5 min after transfection. Blue circles indicate PKR-marked dRIFs detected by the analysis code and yellow boxes indicate detected G3BP1-marked SGs. Distance (pixels) from dRIF to all detected SGs shown below. To quantify proximal formation of G3BP1 puncta, spots within a radius of 100 pixels around detected PKR spots (white dotted circle) are indicated by red bars.

To address the biological consequences of dRIF formation, we first examined the kinetics of dRIF formation relative to activation of PKR and RL. To avoid aberrant PKR activation due to overexpression, we stably introduced mApple-PKR and GFP-G3BP1 into a PKR KO A549 cell line using lentiviral transduction. PKR KO cells expressing mApple-PKR and GFP-G3BP1 did not form spontaneous SGs in the majority of cells (Fig. 5*A*).

Simultaneous live-cell imaging of mApple-PKR, GFP-G3BP1 in PKR KO A549 cells transfected with poly(I:C) showed the formation of both dRIFs and RLBs, which are triggered by RL activation (22), typically within a few minutes of each other (Fig. 5*A*). In cells that formed both dRIFs and RLBs, dRIFs were observed forming before RLBs in 62% of cells, while RLBs were observed to form before dRIFs in 38% of cells (Fig. 5*A*). While the timing of RLB and dRIF formation varied from 5 min to several hours postpoly(I:C) transfection, cells that formed both types of assemblies typically formed both of them within 5 to 10 min of one another. This suggests that the difference in the absolute time to a dsRNA response is likely due to variability in the timing of poly(I:C) release from liposomes. Moreover, these observations suggest that the timing of PKR foci formation and RL activation by poly(I:C) are similar, although they are independent from one another. Line scan analysis revealed that mApple-PKR was neither depleted nor enriched from RLBs, and GFP-G3BP1 was also neither depleted nor enriched in dRIFs (*SI Appendix*, Fig. S2*G*). No instances of cells forming dRIFs, but not RLBs, were observed, validating that dsRNA is necessary for PKR recruitment to foci.

An important question is the timing of dRIF formation relative to PKR activation and translational repression, which can be assessed by the formation of SGs in RL KO A549 cells. For this experiment, we stably introduced mApple-PKR and GFP-G3BP1 into PKR/RL DKO cells (*SI Appendix*, Fig. S2*F*). Strikingly, in cells that formed both dRIFs and SGs upon poly(I:C) transfection, dRIFs formed before SGs in 93% of instances (Fig. 5*B*). This demonstrates that dRIFs typically form before or concurrently with PKR-mediated translation repression. Furthermore, RL KO cells with poly(I:C)-induced SGs stain higher for p-eIF2α, a downstream marker of PKR activation (*SI Appendix*, Fig. S3*A*), than cells without SGs, validating that these cells have undergone PKR-mediated translation repression. In contrast to the results presented in Zappa et al. (24), we do not observe the exclusion of p-eIF2α or eIF2α from dRIFs (*SI Appendix*, Fig. S3 *B* and *C*), allowing for the possibility that PKR within dRIFs can phosphorylate eIF2α.

Consistent with our fixed cell imaging, we observed that mApple-PKR and GFP-G3BP1 formed distinct assemblies upon poly(I:C) transfection, and, generally, neither was depleted nor enriched in the other assembly (Fig. 5*B* and *SI Appendix*, Fig. S2*H*). In 7% of cells, SGs were observed before dRIFs, indicating that visible dRIF formation is not necessary for the formation of PKR-dependent SGs (Fig. 5*B* and *SI Appendix*, Fig. S3*D*). However, we cannot rule out that smaller dRIF assemblies are forming that are below the detection limit of light microscopy, which can happen with RNP condensates (50).

In instances in which dRIFs form near the edge of the cell, we observed that the first SGs form proximally to the dRIF, and then SG formation spreads across the cell (Fig. 5*C*, *SI Appendix*, Fig. S4*A* and S1–S5). This suggests that dRIFs may be sites of PKR activation and that PKR phosphorylates the eIF2α molecules closest to the dRIF first, inhibiting translation locally first. Then, due to the diffusion of p-eIF2α throughout the cell, translation will quickly be inhibited globally across the cell. We interpret this observation to argue that dRIF formation is spatially linked to PKR activation, which argues that dRIFs are involved in PKR activation. However, the possibility that distinct poly(I:C) entry sites into the cell are responsible for the propagation of SG formation across the cell, rather than diffusion of p-eIF2α, cannot be ruled out. In cells where dRIFs form centrally, we observe SGs form concomitantly throughout the cell (*SI Appendix*, Fig. S4*B* and *C*).

RL KO cells will activate GADD34 to resume translation and disassemble SGs after poly(I:C) transfection (21, 22, 51). We observed that dRIFs persisted in cells when SGs were disassembled (Fig. 5*B* and Video S6). This observation argues that dRIF disassembly is not triggered by dephosphorylation of eIF2α or the resumption of active translation. We also observed that approximately one-third of cells formed dRIFs but did not form SGs during the duration of imaging. This shows that PKR recruitment to dRIFs does not always result in the phosphorylation of eIF2α and translational repression (*SI Appendix*, Fig. S3*E*) and raises the question of what factors determine whether a cell that forms dRIFs undergoes translational repression.

In some cells, we observed cells undergoing a round of dRIF and SG formation, followed by SG disassembly, formation of a new dRIF, and a new round of SG formation in the same cell (Video S7). We propose two possible explanations for this observation. After the first round of SG formation, GADD34 may be induced, leading to SG disassembly and resumption of translation. When a second dRIF forms and more PKR is activated, sufficient p-eIF2α is produced to overcome the increased GADD34 levels. Alternatively, PKR within the first dRIF may become inactivated due to some local feedback, which could be a function of dRIF formation, leading to the resumption of translation, which is subsequently inhibited by the formation of a second dRIF. In either case, these multiple rounds of spatially correlating dRIF formation and SG formation provide additional evidence for dRIF formation being linked to PKR activation.

These videos also reveal two properties of dRIFs similar to other biological condensates. First, dRIFs can undergo both fusion and fission (S8 and S9), demonstrating that they share a dynamic consistent assembly mechanism. Second, FRAP analysis of PKR shows that there are both dynamic and mobile pools of PKR in dRIFs, with the majority of PKR being relatively stably associated with dRIFs (Fig. 4*D*) (24).

### PKR Is Not Required for dRIF Formation.

**Fig. 6.**
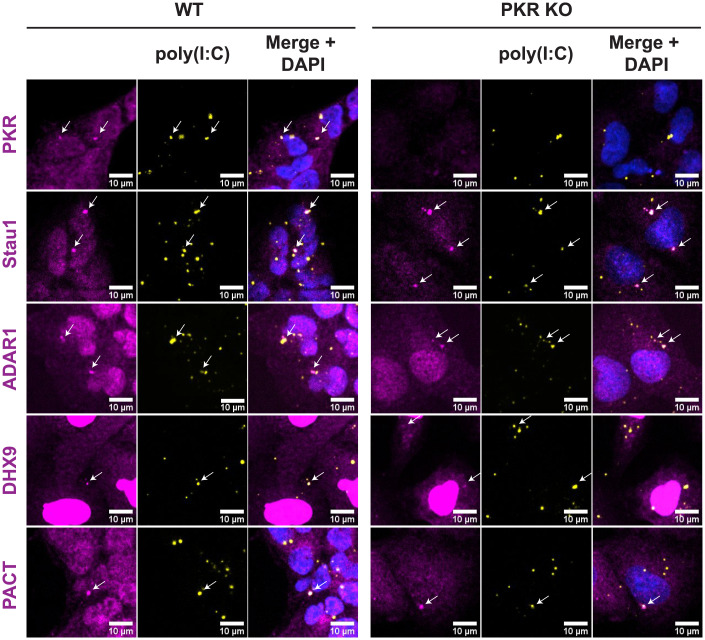
PKR is not required for dRIF formation. IF for PKR, Stau1, ADAR1, DHX9, and PACT in WT and PKR KO A549 cells transfected with labeled poly(I:C) for 4 h. Nuclei shown in blue. White arrows indicate colocalization. (All scale bars, 10 μm.)

An unresolved question is the mechanism by which dRIFs form. One possibility is that the multiple ΔdsRBDs of PKR form multivalent bridges between dsRNA molecules, leading to dRIF condensation. To test this possibility, we stained WT and PKR KO A549 cells for dRIF proteins after poly(I:C) transfection. The localization of ADAR1, PACT, Stau1, and DHX9 to dRIFs were not affected by the loss of PKR (Fig. 6), demonstrating that dRIFs are not simply PKR-dependent assemblies. This suggests that the assembly of dRIFs is driven by other cellular factors, which remain to be identified.

## Discussion

We present several observations documenting that cytosolic dsRNA can trigger the formation of an RNA and protein condensate in human cells referred to as dRIFs. First, we observe that dsRNA forms cytosolic foci following transfection (Fig. 2). Second, those dsRNA foci recruit multiple, but not all, dsRNA-binding proteins, with PKR, ADAR1, PACT, Stau1, NLRP1, and DHX9 showing increased partitioning into dRIFs (Fig. 3). Moreover, dRIFs form in response to increased concentrations of endogenous dsRNA, either due to ADAR1 deficiency or expression of dsRNAs (Fig. 1). dRIFs are distinct from other cytosolic RNP granules and do not generally overlap with SGs, P-bodies, or RLBs (Fig. 1 and *SI Appendix*, Fig. S1). In support of these observations, PKR foci have also been shown to form in response to both poly(I:C) transfection and measles virus infection (24). Although dsRNA foci forming with NLRP6 have been previously described, dRIFs appear to be distinct since we do not observe any recruitment of NLRP6 to dRIFs (*SI Appendix*, Fig. S1).

Several observations argue that dRIFs are formed by dsRNAs serving as a scaffold for proteins with multiple ΔdsRBDs, that can then introduce intermolecular protein–protein interactions between different dsRNA molecules (*SI Appendix*, Fig. S5). First, by using dsRNAs with different fluorescent tags, we demonstrate that dRIFs contain multiple molecules of dsRNA (Fig. 2). Second, longer dsRNAs are more efficient at generating dRIFs than shorter dsRNAs, even at the same mass of dsRNA (Fig. 2). This is consistent with dRIF assembly being promoted by the increased valency of longer RNAs. Furthermore, many dsRNA-binding proteins in dRIFs contain multiple ΔdsRBDs, including Stau1, PACT, PKR, and ADAR1 (Fig. 3). Given this, one possibility is that dRIF assembly is redundant, with any of these proteins providing multivalent dsRNA binding to bridge dsRNA molecules and lead to dRIF formation.

Our data provide several observations suggesting that dRIF formation may modulate the activation of PKR. First, we observe a correlation between cells that form visible dRIFs and those that trigger eIF2α phosphorylation and translation repression, as evidenced by SG formation (Figs. 1 and 5). Second, in cells lacking RNase L, which prevents RL-dependent eIF2α phosphorylation (21), we observe that dRIFs form before SGs more than 90% of the time (Fig. 5). This argues that dRIF formation precedes translation repression and may contribute to PKR activation. Third, in cells where dRIFs form near the edge of the cell, we observe that SGs first form close to the dRIF before forming elsewhere throughout the cell. This demonstrates a spatial correlation between dRIF formation and PKR activation, which is consistent with dRIFs serving as sites of PKR activation (Fig. 5*C* and *SI Appendix*, Fig. S4*A* and S1–S5 and S7). We anticipate that smaller dRIF assemblies form that we cannot observe in the light microscope and contribute to PKR activation because in ∼7% of RL KO cells, we observed that SGs formed before dRIFs were observed, which could be due to smaller dRIFs forming, activating PKR, and then merging into a dRIF large enough to be visualized at a later time (*SI Appendix*, Fig. S3*D*).

Other observations have been used to suggest that dRIFs could be inhibitory to PKR activation (24). First, Zappa et al. observed that eIF2α is not enriched in dRIFs, which they suggest may limit activated PKR molecules in dRIFs interacting with its substrate. Both we and Zappa et al. demonstrate that PKR in dRIFs is fairly immobile, and therefore if PKR is unable to interact with eIF2α while in a dRIF, then the dRIF will act as a sink for PKR signaling. Second, mutations that alter PKR-PKR interactions and decrease PKR recruitment to dRIFs have been suggested to enhance p-eIF2α in response to poly(I:C) (24).

Future experiments will need to more fully address the role of dRIF formation in the control of PKR function. One possibility is that dRIF formation has different effects on PKR depending on the rate-limiting step in the activation of PKR and its interaction with the eIF2α substrate. For example, under low concentrations of dsRNA, the formation of dRIFs could create a higher local concentration of dsRNA to trigger PKR autophosphorylation. Alternatively, when dsRNA is high, dRIFs may create an excess of stable binding sites for PKR that limits the accessibility of PKR to the eIF2α substrate. Finally, the formation of dRIFs may facilitate possible negative feedback loops to down-regulate PKR activation. Understanding the driving mechanisms of dRIF formation that enhance or limit their formation may shed light on the relationship between dRIF formation and PKR signaling.

The formation of dRIFs has implications for the activation of PKR in a number of biological contexts. We anticipate that dRIFs may be important for PKR activation in some viral infections since viruses use multiple mechanisms to limit PKR activation (reviewed in Cesaro and Michiels [52]). For example, hepatitis C virus blocks PKR dimerization through the NS5A protein (53, 54). Given this, we hypothesize that the ability of cells to create a high local concentration of PKR in dRIFs will limit the ability of the NS5A protein to prevent PKR activation. Finally, we anticipate that dRIFs will be important in any biological context in which the amount of dsRNA is limiting. These biological contexts include the initial phases of a viral infection, the higher levels of endogenous dsRNA in some Aicardi-Goutières syndromes (55), and even in neurological diseases in which the expression of repeat expansion RNAs with dsRNA-like character can lead to the activation of PKR (56, 57). Consistent with a role for dRIFs in neurological disease, cells expressing the G_4_C_2_ repeat in the *C9orf72* gene that can cause ALS show activation of PKR and concentration of PKR into discreet cytoplasmic foci that we suggest are dRIFs (58). Given these roles, an understanding of dRIF formation, the mechanisms by which dRIFs influence human disease, and how their manipulation may be therapeutic will be important areas of research.

The discovery of dRIFs adds to the growing set of observations whereby activation of the innate immune system through the recognition of either dsRNA or cytosolic DNA involves the formation of a nucleic acid–protein condensate. For example, the recognition of dsRNA by NLRP6 involves the formation of dsRNA-NLRP6 condensates (26), and our data suggest that dRIFs may play a role in activating PKR and NLRP1 in response to dsRNA. Similarly, the recognition of cytosolic DNA by cyclic guanosine monophosphate-adenosine monophosphate synthase (cGAS) involves the formation of a DNA–protein condensate (59). Thus, the formation of condensates that concentrate both the nucleic acid and the sensor provides a useful mechanism to increase the sensitivity of the innate immune response to nucleic acid.

## Materials and Methods

Cell lines were maintained at 5% CO_2_ at 37 °C in Dulbecco’s modified Eagle’s medium supplemented with 10% fetal bovine serum and 1% penicillin/streptomycin. Stable cell lines were generated using lentiviral transduction. IF was performed as described by Corbet et al. (28). A complete description of the materials and methods can be found in the *SI Appendix, SI Materials and Methods*.

## Supplementary Material

Supplementary File

Supplementary File

Supplementary File

Supplementary File

Supplementary File

Supplementary File

Supplementary File

Supplementary File

Supplementary File

Supplementary File

## Data Availability

All of the study data are included in the article and/or *SI Appendix*.
