# Supplementary material for: dsRNA-induced condensation of antiviral proteins modulates PKR activity
Source: Proc Natl Acad Sci U S A. 2022 Aug 8;119(33):e2204235119. doi: 10.1073/pnas.2204235119 (PMC9388085; doi:10.1073/pnas.2204235119)
Supplement: Supplementary File [file pnas.2204235119.sapp.pdf]

SUPPLEMENTAL FIGURE 1

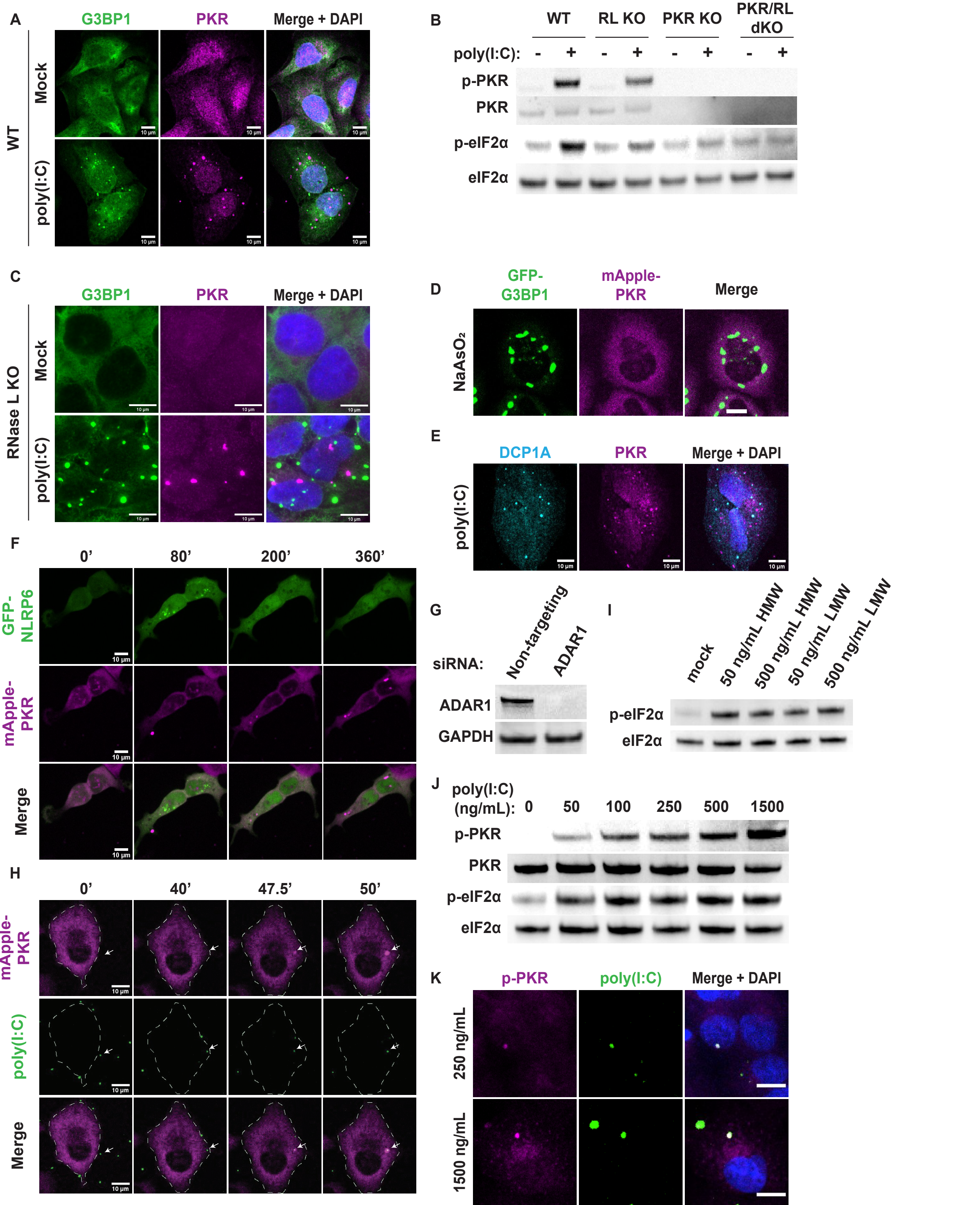

SUPPLEMENTAL FIGURE 2

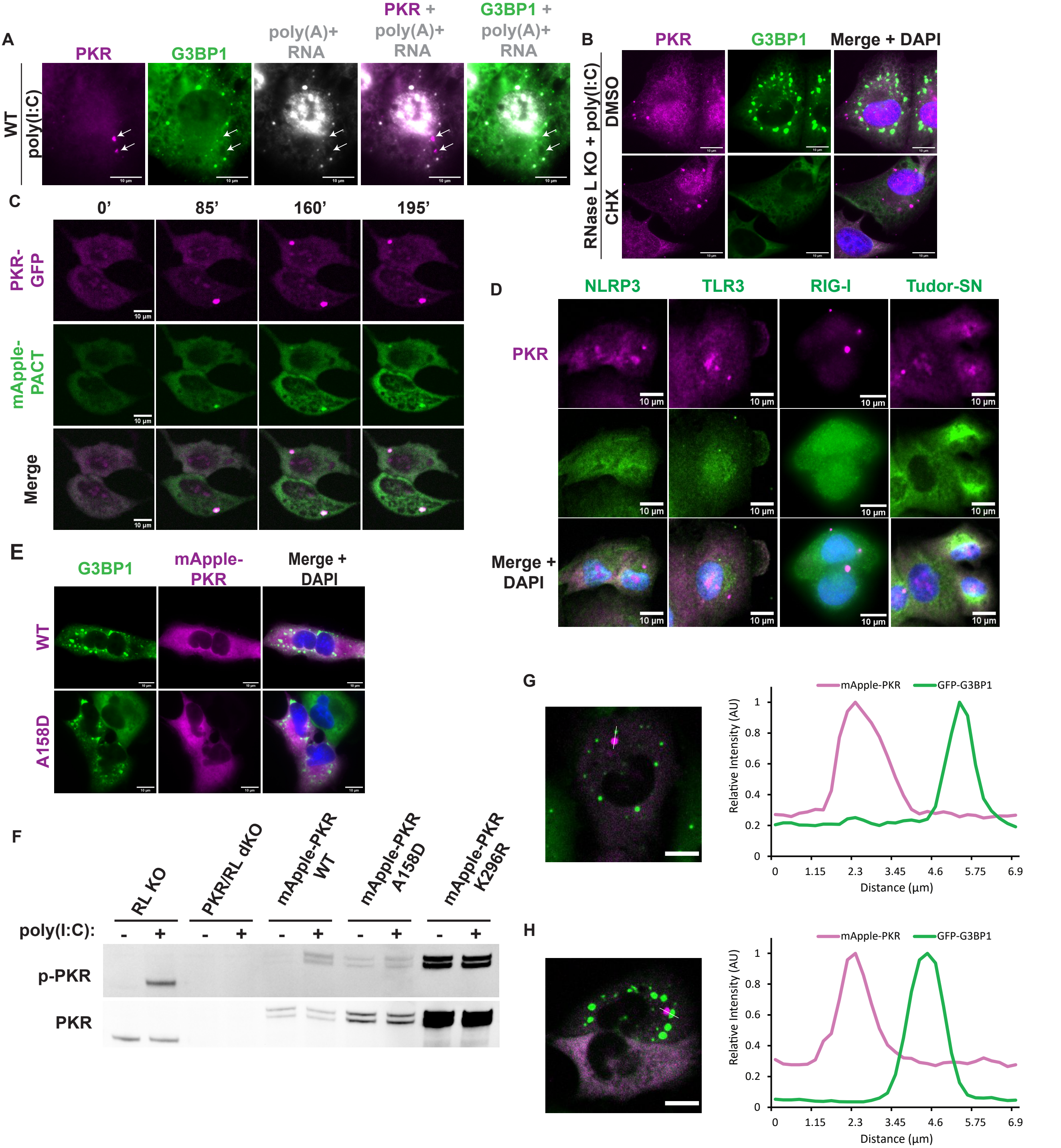

SUPPLEMENTAL FIGURE 3

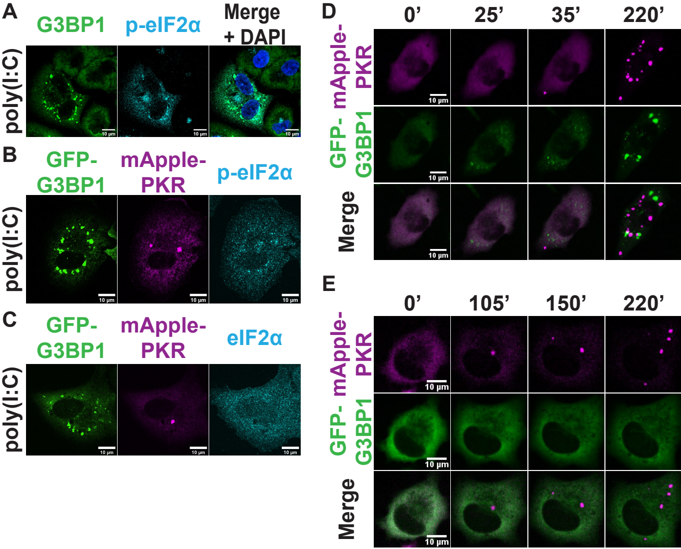

SUPPLEMENTAL FIGURE 4

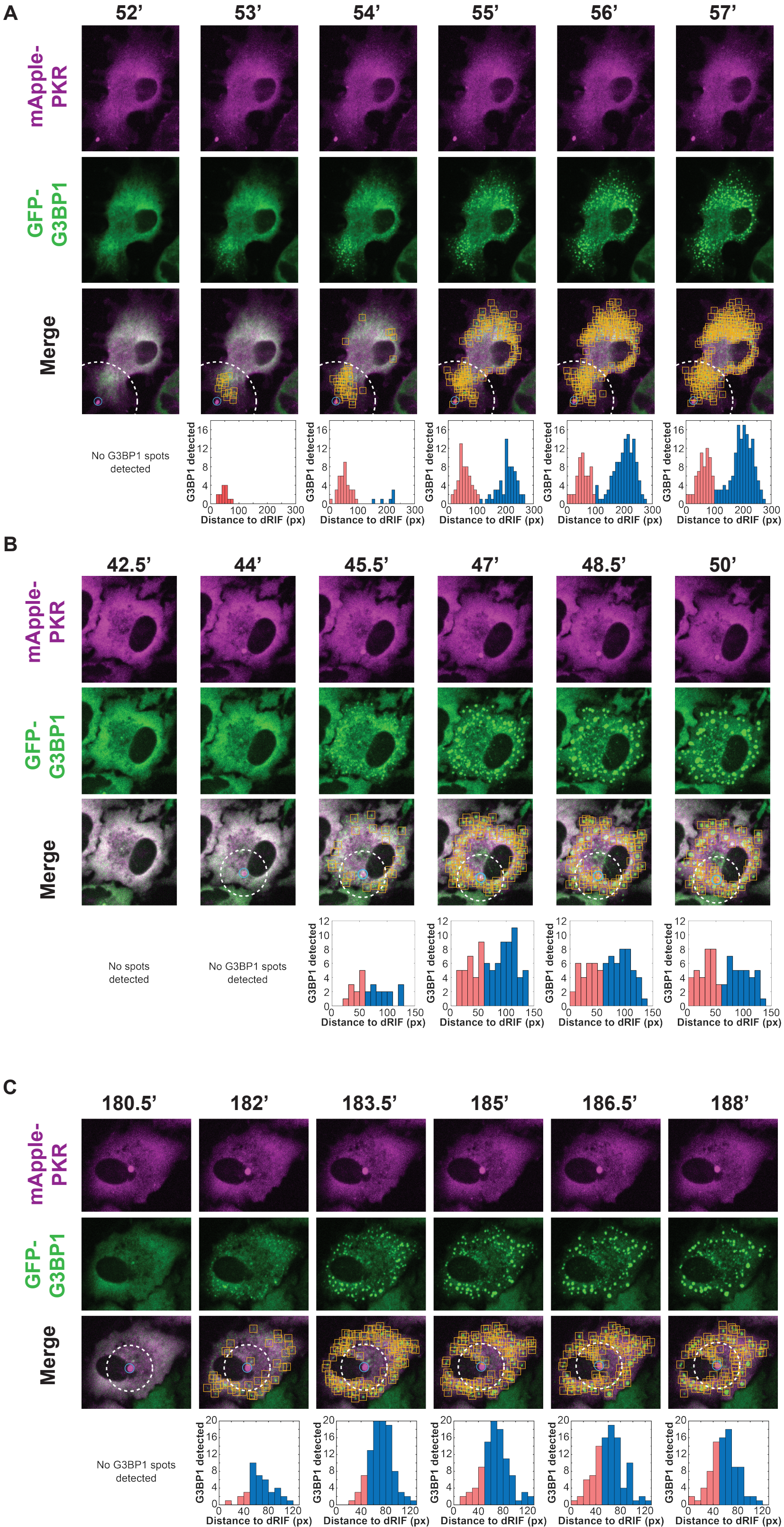

SUPPLEMENTAL FIGURE 5

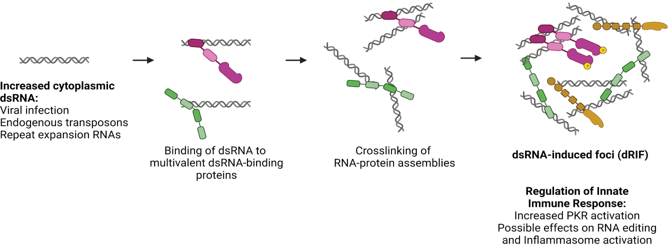

Supplemental Figure Legends

Supplemental Figure 1. A) IF for PKR and G3BP1 in WT U-2 OS cells transfected with poly(I:C). Nuclei shown in blue. B) Western blot for phospho-PKR (p-PKR), PKR, p-eIF2α, eIF2α in WT, RL KO, PKR KO, and PKR/RL dKO A549 cells transfected with 500 ng/mL poly(I:C). C) IF for PKR and G3BP1 in RL KO U-2 OS cells transfected with poly(I:C). D) PKR KO A549 cells expressing GFP-G3BP1 and mApple-PKR treated with 500 μM $NaAsO_2$ for 45 minutes. E) IF for DCP1A and PKR in WT U-2 OS cells transfected with poly(I:C). F) PKR KO A549 cells expressing mApple-PKR and GFP-NLRP6 transfected with 500 ng/mL poly(I:C) and imaged 0-, 80-, 200-, and 360-minutes after transfection. G) Western blot for ADAR1 and GAPDH in U-2 OS cells treated with non-targeting or ADAR1 siRNA. H) PKR KO A549 cells expressing mApple-PKR and transfected with labeled poly(I:C) and imaged at 0-, 40-, 47.5-, and 50-minutes after transfection. I) Western Blot of p-eIF2α and eIF2α in RL KO A549 cells transfected with mock, 50 ng/mL, and 500 ng/mL high molecular weight (HMW) or low molecular weight (LMW) poly(I:C) for 4 hours. J) Western Blot of p-PKR, PKR, p-eIF2α, and eIF2α in RL KO A549 cells transfected with mock, 50, 100, 250, 500 and 1500 ng/mL poly(I:C) for 4 hours. K) IF for p-PKR in RL KO A549 cells transfected with 250 or 1500 ng/mL fluorescently labeled poly(I:C) for 4 hours. All scale bars, 10 μm.

Supplemental Figure 2. A) IF for PKR and G3BP1, FISH for poly(A)+ RNA in A549 cells transfected with poly(I:C). White arrows indicate location of dRIFs. Scale bars, 10 μm. B) IF for PKR and G3BP1 in RL KO A549 cells treated with DMSO or 10 ug/mL cycloheximide (CHX) and transfected with 500 ng/mL poly(I:C) for 2 hours. Scale bars, 10 μm. C) PKR KO A549 cells expressing mApple-PACT and PKR-GFP transfected with poly(I:C) and imaged at 0-, 85-, 160-, and 195-minutes after transfection. D) IF for PKR and dsRNA-binding proteins NLRP3, TLR3, RIG-I, and Tudor-SN in A549 cells transfected with poly(I:C) for 4 hours. E) WT A549 cells transiently transfected with mApple-PKR WT or A158D and stained for G3BP1. F) Western blot for p-PKR and PKR in RL KO, PKR/RL dKO, and PKR/RL dKO A549 cells expressing mApple-PKR WT, A158D, or K296R. G) Line-scan of PKR KO A549 cells expressing mApple-PKR and GFP-G3BP1 after poly(I:C) transfection. Maximum intensity in the line scan of each channel set to 1. H) Line-scan of PKR/RNase L dKO A549 cells expressing mApple-PKR and GFP-G3BP1 after poly(I:C) transfection. Maximum intensity in the line scan of each channel set to 1.

Supplemental Figure 3. A) IF for G3BP and p-eIF2α in RL KO A549 cells transfected with poly(I:C) for 4 hours. B) IF for p-eIF2α in PKR/RL dKO cells expressing mApple-PKR and GFP-G3BP1 and transfected with poly(I:C) for 4 hours. C) IF for eIF2α in PKR/RL dKO cells expressing mApple-PKR and GFP-G3BP1 and transfected with poly(I:C) for 4 hours. D) Images demonstrating SGs forming prior to dRIFs in PKR/RNase L dKO A549 cells expressing GFP-G3BP1 and mApple-PKR transfected 500 ng/mL poly(I:C) and imaged at 0-, 25-, 35-, and 220-minutes after transfection. E) Images demonstrating dRIFs forming without SGs in PKR/RL dKO A549 cells expressing GFP-G3BP1 and mApple-PKR transfected 500 ng/mL poly(I:C) and imaged at 0-, 105-, 150-, 220-minutes after transfection. All scale bars, 10 μm

Supplemental Figure 4. A) Images showing asymmetric SG formation proximal to dRIF in PKR/RNase L double KO A549 cells expressing GFP-G3BP1 and mApple-PKR transfected with 500 ng/mL poly(I:C) and imaged at 52-, 53-, 54-, 55-, 56-, and 57-minutes after transfection. Distance (pixels) from dRIF to all detected stress granules shown below. B) Images showing SG formation after dRIF formation in PKR/RNase L double KO A549 cells expressing GFP-G3BP1 and mApple-PKR transfected with 500 ng/mL poly(I:C) and imaged at 42.5-, 44-, 45.5-, 47, 48.5-, and 50-minutes after transfection. Distance (pixels) from dRIF to all detected stress granules shown below. C) Images showing SG formation after dRIF formation in PKR/RNase L double KO A549 cells expressing GFP-G3BP1 and mApple-PKR transfected with 500 ng/mL poly(I:C) and imaged at 42.5-, 44-, 45.5-, 47-, 48.5-, and 50-minutes after transfection. Distance (pixels) from dRIF to all detected stress granules shown below. In all subfigures, the blue circles indicate detected dRIFs and yellow boxes indicate detected stress granules. To quantify proximal formation of stress granules, spots within a region around the dRIFs, as indicated by the white dotted circles, were counted and are indicated by red bars in the histograms. The radius of the circle were either 100 pixels (A) or 50 pixels (B and C), chosen relative to the size of the cell.

Supplemental Figure 5. Model for dRIF formation. Under conditions of increased cytoplasmic dsRNA such as during viral infection, dsRNA-binding proteins bind to dsRNA. Multivalent protein-RNA and protein-protein interactions crosslink these RNA-protein assemblies, leading to the formation of a dsRNA-induced foci (dRIF), which results in increased PKR activation and may also have implications for RNA editing by ADAR1 and inflammasome activation by NLRP1. Created with BioRender.com

Video S1-S5. Video showing asymmetrical formation of GFP-G3BP1 stress granules (green) near site of mApple-PKR foci (magenta) and spreading across cell. Scale bar, 10 μm. Time since poly(I:C) transfection indicated.

Video S6. Video showing persistence of mApple-PKR foci (magenta) after dissolution of GFP-G3BP1 stress granules (green) formed after poly(I:C) transfection. Scale bar, 10 μm. Time since poly(I:C) transfection indicated.

Video S7. Video showing a cell undergo two rounds of dRIF and stress granule formation after poly(I:C) transfection. Scale bar, 10 μm. Time since poly(I:C) transfection indicated.

Video S8. Video showing fusion of mApple-PKR foci formed after poly(I:C) transfection. Scale bar, 10 μm. Time since poly(I:C) transfection indicated.

Video S9. Video showing fission of mApple-PKR foci formed after poly(I:C) transfection. Scale bar, 10 μm. Time since poly(I:C) transfection indicated.

SI Materials and Methods

Cell Culture and Drug Treatment

The parental A549 cell line was provided by Dr. Christopher Sullivan (1). The RNase L KO, PKR KO, and PKR/RNase L double KO A549 and the RNase L KO U-2 OS cell lines were generated as described in (2,3). The sgRNAs for KO cell lines used are as follows: PKR_sgRNA_1_sense, CACCGATTCAGGACCTCCACATGAT; PKR_sgRNA_1_antisense, CATCATGTGGAGGTCCTGAATCAAA; PKR_sgRNA_2_sense, CACCGTTATCCATGGGGAATTACAT; and PKR_sgRNA_2_antisense, CATGTAATTCCCCATGGATAACAAA, RL_sgRNA_1_sense, CACCGcgcatctgctgctggacca; RL_sgRNA_1_antisense, AAACtggtccagcagcagatgcgC. The WT U-2 OS cell line was provided by Dr. Nancy Kedersha and Dr. Paul Anderson (4,5). All cell lines were maintained at 5% CO_2 at 37 °C in Dulbecco's Modified Eagle Medium (DMEM) supplemented with 10% fetal bovine serum (FBS) and 1% penicillin/streptomycin. Cells were routinely tested for mycoplasma contamination and were negative.

Plasmids

pGW1-mApple was a gift from Dr. Sami Barmada (6). pGW1-mApple-PKR was generated from pGW1-mApple and pPET-PKR/PPase (Addgene Plasmid #42934) using In-Fusion® Snap Assembly (Takara Bio # 638947) and the following primers: GACGAGCTGTACAAGatggctggtgatctttcagca and GATCCGGTGGATCCCCTAACATGTGTGTCGTTCATTTTTC. pGW1-mApple was linearized with SmaI (NEB # R0141S).

pGW1-mApple-PKR-K296R was generated from pGW1-mApple-PKR using Agilent QuikChange II XL Site-Directed Mutagenesis Kit (Neta Scientific # 200521) using the following primers: gttattatatttaacacgtctaataacgtaagtctttccgtcaattctgtgttttg and caaaacacagaattgacggaaagacttacgttattagacgtgttaaatataataac.

pLV-EF1-mApple-PKR WT and K296R were generated from pLV-EF1-Cre-PGK-Puro (Addgene Plasmid #108543) and pGW1-mApple-PKR using NEBuilder® HiFi DNA Assembly (NEB #E5520) and the following primers: gcaggctgccaccatgGTGAGCAAGGGCGAG and acaagaaagctgggtctaacatgtgtgtcg. pLV-EF1-Cre-PGK-Puro was linearized with BamHI (NEB #R0136S).

pLV-EF1-mApple-PKR A158D was generated from pLV-EF1-mApple-PKR WT using Agilent QuikChange II XL Site-Directed Mutagenesis Kit and the following primers: tctgaagatatgcaagtttatcggccaattgttttgcttcc and ggaagcaaaacaattggccgataaacttgcatatcttcaga.

pPKR-GFP was generated from pGW1-mApple-PKR and pmGFP (Addgene # 117926) using NEBuilder® HiFi DNA Assembly with the following primers: ggagacccaagctggatggctggtgatctt and gcccttgctcaccatacatgtgtgtcgttc. pmGFP was linearized with NheI (NEB # R0131)

pLV-EF1-PKR-GFP was generated from pPKR-GFP and pLV-EF1-Cre-PGK-Puro using NEBuilder® HiFi DNA Assembly (NEB #E5520) and the following primers: gcaggctgccaccatggctggtgatctttca and acaagaaagctgggtctacttgtacagctc. pLV-EF1-Cre-PGK-Puro was linearized with BamHI (NEB #R0136S).

pGW1-mApple-PACT was generated from pcDNA-PACT (Addgene #15667) and pGW1-mApple-ADAR1p150 using NEBuilder® HiFi DNA Assembly with the following primers: GACGAGCTGTACAAGATGTCCCAGAGCAGG and GATCCGGTGGATCCCCTACTTTCTTTCTGCTAT. pGW1-mApple-ADAR1p150 was linearized with SwaI (NEB # R0604S).

pmGFP-NLRP6 was generated from pmGFP and NLRP6 cDNA ORF Clone, Human, untagged (Sino Biological # HG21799-UT) using NEBuilder® HiFi DNA Assembly with the following primers: catggacgagctgtacaagATGGACCAGCCAGAG and gtttaaacgggccctctagaTCAGAAGGTCGAGATG. pmGFP was linearized with NheI (NEB # R0131).

pEGFP-IRAlus-Lin28 was a gift from Gordon Carmichael & Ling-Ling Chen (Addgene plasmid # 92347 ; http://n2t.net/addgene:92347 ; RRID:Addgene_92347).

pGW1-mApple-ADAR1p150 WT, ΔdsRBD, and dsRBD were generated as described in (7).

siRNA Knockdowns and Plasmid Transfections

siRNA knockdowns were performed using Lipofectamine RNAiMAX (Thermo Fisher Cat # 13778075) according to Manufacturer's protocol for 48 hours. The following siRNA was used: ADAR1 (Thermo Fisher Cat # 4390824), Silencer™ Negative Control No. 1 siRNA (Non-targeting) (Thermo Fisher Cat # AM4611)

Plasmids were transfected using jetPRIME® transfection reagent (VWR Scientific Cat # 89129-920) or Lipofectamine™ 2000 Transfection Reagent (Thermo Fisher Scientific #11668027) according to manufacturer's protocol. Cell media was replaced 2 hours post-transfection. Cells were imaged or fixed 24 hours post transfection.

Drug Treatments

Sodium arsenite (Millipore Sigma, S7400) was diluted in UltraPure DNase/RNase-Free Distilled Water (Thermo Fisher Scientific, 10977-023). Treatment was done for 1 hr at 37 °C. C16 (Imidazolo-oxindole PKR inhibitor C16,≥98% (HPLC), Sigma-Aldrich #I9785-5MG) was prepared in DMSO and treatments were done for 24 hours at 37 °C. Poly(I:C) (InvivoGen tlrl-pic, tlrl-picf, tlrl-picr, tlrl-picw) was resuspended in UltraPure DNase/RNase-Free Distilled Water and transfected into cells using Lipofectamine 2000 reagent (Thermo Fisher Scientific, 11668030) according to the manufacturer's protocol. 3 μl Lipofectamine was used for 1 μg poly(I:C). Poly(I:C) treatments were done for 4 hours unless otherwise stated in figure legend. Cycloheximide (Sigma-Aldrich, C7698-1G) was prepared in DMSO, and treatment was performed for 2 hrs.

Lentiviral Transductions

Lentivirus was generated in HEK293T cells. HEK293T cells in T-25 flask were transfected with 2 μg pLV-mApple-PKR (WT, A158D, or K296R), pLV-PKR-GFP, or pLenti–EF1–GFP–G3BP1–blast (3), 1 μg pRSV-Rev, 1 μg pVSV-G, and 1 μg of pMDLg–pRRE and 15 μL Lipofectamine 2000. Media was collected 48 hours post-transfection and filtered through a 0.45 μm filter. To generate stable cell lines, A549 cells were incubated 1 mL of lentivirus stock containing 10 μg/mL polybrene for 1 hour, at which point fresh media was added to the cells. 48

hours post-transduction, cells were selected with 1.5 μg/mL puromycin and/or blasticidin-containing media. Fluorescent cells were also selected for using fluorescence activated cell sorting (FACS).

Immunofluorescence

Immunofluorescence was performed as described in (7). The following antibodies were used at 1:500 dilutions: G3BP1 antibody (Abcam ab56574), ADAR1 (abcam ab88574), PACT (Abcam ab31967), PKR (Cell Signaling Technology #122976), PKR (Santa Cruz Biotechnology #sc-6282), Stau1 (Novus Biologicals #NBP1-33202), DHX9 (Proteintech C827D22), DCP1A (Millipore Sigma WH0055802M6-50UG), DCP1B (Cell Signaling Technology #13233S), TLR3 (Abcam ab137722), NLRP1 (Abcam ab36852), NLRP3 (Thermo Scientific PA5-109211), phospho-PKR (Thermo Scientific # MA5-32086). The following antibodies were used at 1:1000 dilutions: K1 (Cell Signaling Technology #28764S), p-eIF2α (Cell Signaling Technology #9721S) and eIF2α (Cell Signaling Technology #9722S). The following secondary antibodies were used at 1:1000: Goat anti-rabbit (abcam ab6717), Goat anti-rabbit (abcam ab150079), Goat anti-mouse (abcam ab150115), Goat anti-mouse (abcam ab6785), Donkey anti-rabbit (abcam ab150062).

Sequential Immunofluorescence and FISH

Sequential immunofluorescence and FISH was performed as described in (8). Poly d(T) probes were ordered from Integrated DNA Technologies as 30 deoxythymidines with a 5' Cy5 modification.

SDS-PAGE and Western Blotting

SDS-PAGE and Western blotting was performed as described in (7). The following antibodies were used at 1:1000 in TBS-T containing 5% BSA: p-eIF2α (Cell Signaling Technology #9721S), eIF2α (Cell Signaling Technology #9722S) PKR (Cell Signaling Technology # 12297S), phospho-PKR (Thermo Scientific # MA5-32086), ADAR1 (Abcam # ab126745), GAPDH-HRP (Santa Cruz Biotechnology # sc-47724 HRP). The anti-rabbit IgG, HRP-linked antibody (Cell Signaling 7074S) was used at 1:2000 in TBS-T containing 5% BSA. Membranes were stripped with Restore Western Blot Stripping Buffer (Thermo Scientific #21059) for 15-30 minutes at room temperature.

Microscopy and Image Analysis

A549 cells were grown in 24-well glass plates (Cellvis #P24-1.5H-N) or on coverslips (Thomas Scientific # 1217N79). Live cell imaging was performed on a Nikon Spinning Disc Confocal microscope with a 2x Andor Ultra 888 EMCCD camera and a 40x air objective. Fixed cells were imaged on a widefield DeltaVision Elite microscope with a 100X oil objective and PCO Edge sCMOS camera or the Nikon Spinning Disc Confocal microscope with a 2x Andor Ultra 888 EMCCD camera and a 40x air or 100x oil objective. 7-20 z slices were imaged at 0.2 μm (DeltaVision or Spinning Disc 100x) or 0.6 μm (Spinning Disc 40x) distance between slices. All images shown are of a single z plane unless otherwise specified in figure legend.

dRIF enrichment was calculated using ImageJ. Maximum intensity projections of all z slices were generated, a line was drawn through the dRIF and through the cytoplasm, and the average intensity of each line was calculated. Enrichment was calculated as dRIF intensity/cytoplasm intensity. dRIF and cytoplasm volume

were calculated using Bitplane Imaris software as described in (7,9) for stress granule quantification. Statistical analysis was performed using an unpaired two-tailed t-test. For $p > 0.05$, analyses were designated as not significant.

A custom MATLAB script was developed to analyze the resulting images. The script identified cells and both fluorescently labeled PKR and G3BP1 puncta, tracked individual PKR spots over time, and measured the distance between PKR and G3BP1 puncta. The datasets were initially preprocessed to calculate the maximum intensity projection (MIP) for each image.

To identify individual cells, the mApple and GFP MIP images were summed, then an intensity threshold was manually chosen to distinguish the fluorescent cells from the dark background. This process yielded a binary mask, where each pixel in the image that belonged to a cell was labeled true and every other pixel was labeled false. The watershed algorithm was then employed to separate touching cells. As the cells were occasionally confluent or had protrusions, the binary mask was manually edited using ImageJ/Fiji to correct mislabeled pixels as necessary.

To identify the puncta in the mApple (corresponding to dRIFs) and the GFP (corresponding to stress granules) channels, each MIP image was first blurred using a Gaussian filter with a standard deviation of 0.5 to smooth the image and remove any “hot” pixels. The background signal within each cell was then subtracted by applying a morphological top hat filter, with a 2-pixel disk shaped structuring element. The final background subtracted image was then subjected to an intensity threshold to obtain a mask identifying each spot. Additionally, we used the edited cell masks to exclude any spots that were found outside of the cells of interest.

Finally, a previously developed tracking algorithm (10) was then used to track the identified PKR puncta over the course of the movie. For each frame in which both PKR and G3BP1 puncta existed, the algorithm also computed the straight-line distance between the PKR and GFP spots. The resulting data was then analyzed to generate the movie files, static images, and histograms, as shown in the results. The code used to analyze the images in this publication is available for download at https://github.com/jwtay1/tracking-stress-granules/.

FRAP

A549 cells were grown in 24-well glass plates (Cellvis #P24-1.5H-N). Cell media was replaced with OptiMEM media immediately prior to imaging. FRAP was done on a Nikon A1R Laser Scanning Confocal microscope as follows: acquire 2 images 5 second apart prior to bleaching, bleach for 9.54 seconds with 405 and 488 nm lasers at 100% laser power, image every 5 seconds second for 1 minute during recovery. Mean intensity within the bleached area was determined using Nikon Elements software. Intensities were normalized to the average intensity of the region of interest (ROI) at time 0 (set to 1) and at the first time-point post-bleaching (set to 0). Graphs represent averages of three independent experiments where at least 10 foci were bleached for each experiment. Error bars represent standard deviation.

SI Appendix References

1. Burke JM, Kincaid RP, Nottingham RM, Lambowitz AM, Sullivan CS. DUSP11 activity on triphosphorylated transcripts promotes Argonaute association with noncanonical viral microRNAs and regulates steady-state levels of cellular noncoding RNAs. Genes Dev 2016;30:2076–2092.

2. Burke JM, Moon SL, Matheny T, Parker R. RNase L Reprograms Translation by Widespread mRNA Turnover Escaped by Antiviral mRNAs. Molecular Cell 2019;75:1203-1217.e5.

3. Burke JM, Lester ET, Tauber D, Parker R. RNase L promotes the formation of unique ribonucleoprotein granules distinct from stress granules. J Biol Chem 2020;295:1426–1438.

4. Kedersha N, Panas MD, Achorn CA, Lyons S, Tisdale S, Hickman T, Thomas M, Lieberman J, McInerney GM, Ivanov P, Anderson P. G3BP-Caprin1-USP10 complexes mediate stress granule condensation and associate with 40S subunits. J Cell Biol 2016;212:845–860.

5. Kedersha N, Tisdale S, Hickman T, Anderson P. Real-time and quantitative imaging of mammalian stress granules and processing bodies. Methods Enzymol 2008;448:521–552.

6. Barmada SJ, Serio A, Arjun A, Bilican B, Daub A, Ando DM, Tsvetkov A, Pleiss M, Li X, Peisach D, Shaw C, Chandran S, Finkbeiner S. Autophagy induction enhances TDP43 turnover and survival in neuronal ALS models. Nat Chem Biol 2014;10:677–685.

7. Corbet GA, Burke JM, Parker R. ADAR1 limits stress granule formation through both translation-dependent and translation-independent mechanisms. Journal of Cell Science 2021;134. Available at: https://doi.org/10.1242/jcs.258783. Accessed September 7, 2021.

8. Khong A, Jain S, Matheny T, Wheeler JR, Parker R. Isolation of mammalian stress granule cores for RNA-Seq analysis. Methods 2018;137:49–54.

9. Khong A, Matheny T, Jain S, Mitchell SF, Wheeler JR, Parker R. The Stress Granule Transcriptome Reveals Principles of mRNA Accumulation in Stress Granules. Molecular Cell 2017;68:808-820.e5.

10. Hill NC, Tay JW, Altus S, Bortz DM, Cameron JC. Life cycle of a cyanobacterial carboxysome. Sci Adv 2020;6:eaba1269.
